# Soluble receptor for advanced glycation end products (sRAGE) is associated with obesity rates: a systematic review and meta-analysis of cross-sectional study

**DOI:** 10.1186/s12902-023-01520-1

**Published:** 2023-12-15

**Authors:** Nahla A Tayyib, Pushpamala Ramaiah, Shadia Hamoud Alshahrani, Ria Margiana, Sami G. Almalki, A. K. Kareem, Rahman S. Zabibah, Abdullah M. Shbeer, Saad Hayif Jasim Ali, Yasser Fakri Mustafa

**Affiliations:** 1 Vice Deanship, Postgraduate Research and Scientific Studies, Faculty of Nursing, Umm Al-Qura University, Makkah, Saudi Arabia; 2 Faculty of Nursing, Umm al- Qura University, Makkah, Saudi Arabia; 3 Medical Surgical Nursing Department, King Khalid University, Khamis Mushate, Saudi Arabia; 4 Department of Anatomy, Faculty of Medicine, Universitas Indonesia, Jakarta, Indonesia; 5 Master’s Programme Biomedical Sciences, Faculty of Medicine, Universitas Indonesia, Jakarta, Indonesia; 6 Andrology Program, Faculty of Medicine, Universitas Airlangga, Surabaya, Indonesia; 7 Dr. Soetomo General Academic Hospital, Surabaya, Indonesia; 8 Department of Medical Laboratory Sciences, College of Applied Medical Sciences, Majmaah University, Majmaah, 11952 Saudi Arabia; 9 Biomedical Engineering Department, Al-Mustaqbal University College, Babylon, Iraq; 10 Medical Laboratory Technology Department, College of Medical Technology, The Islamic University, Najaf, Iraq; 11 Department of Surgery, Faculty of Medicine, Jazan University, Jazan, Saudi Arabia; 12 Department of medical laboratory, College of Health and Medical Technololgy, Al-Ayen University, Thi-Qar, Iraq; 13 Department of Pharmaceutical Chemistry, College of Pharmacy, University of Mosul, Mosul, 41001 Iraq

**Keywords:** sRAGE, Obesity, Adult, Meta-analysis, Systematic review

## Abstract

**Background:**

Several studies have highlighted the possible positive effects of soluble receptor for advanced glycation end products (sRAGE) against obesity. However, due to their inconsistent results, this systematic review and meta-analysis aimed to quantitatively evaluate and critically review the results of studies evaluating the relationship between sRAGE with obesity among adult population.

**Methods:**

In the systematic search, the eligibility criteria were as follows: studies conducted with a cross-sectional design, included apparently healthy adults, adults with obesity, or obesity-related disorders, aged over 18 years, and evaluated the association between general or central obesity indices with sRAGE.

**Results:**

Our systematic search in electronic databases, including PubMed, Scopus, and Embase up to 26 October, 2023 yielded a total of 21,612 articles. After removing duplicates, screening the titles and abstracts, and reading the full texts, 13 manuscripts were included in the final meta-analysis. According to our results, those at the highest category of circulating sRAGE concentration with median values of 934.92 pg/ml of sRAGE, had 1.9 kg/m^2^ lower body mass index (BMI) (WMD: -1.927; CI: -2.868, -0.986; *P* < 0.001) compared with those at the lowest category of sRAGE concentration with median values of 481.88 pg/ml. Also, being at the highest sRAGE category with the median values of 1302.3 pg/ml sRAGE, was accompanied with near 6 cm lower waist circumference (WC) (WMD: -5.602; CI: -8.820, -2.383; *P* < 0.001 with 86.4% heterogeneity of I^2^) compared with those at the lowest category of sRAGE concentration with median values of 500.525 pg/ml. Individuals with obesity had significantly lower circulating sRAGE concentrations (WMD: -135.105; CI: -256.491, -13.72; *P* = 0.029; with 79.5% heterogeneity of I^2^). According to the subgrouping and meta-regression results, country and baseline BMI were possible heterogeneity sources. According to Begg’s and Egger’s tests and funnel plots results, there was no publication bias.

**Conclusion:**

According to our results, higher circulating sRAGE concentrations was associated with lower BMI and WC among apparently healthy adults. Further randomized clinical trials are warranted for possible identification of causal associations.

**Supplementary Information:**

The online version contains supplementary material available at 10.1186/s12902-023-01520-1.

## Introduction

Advanced glycation end-products (AGEs) are a group of compounds formed by non-enzymatic glycation of lipids, proteins, and nucleic acids [1, 2]. AGEs have two endogenous and exogenous sources, while the first is formed during normal metabolism in body and the second is derived from foods or tobacco smoke [3]. AGEs are potential ingredients that lead to oxidative stress and chronic inflammation; they act through binding to their receptors like receptor for advances glycation end products (RAGE) [4]. RAGE is present in numerous differentiated adult cell types, including immune and endothelial, and is a multi-ligand receptor belonging to immunoglobulin superfamily [5–7]. AGE binding to its receptor, RAGE, triggers the cell signaling pathways’ activation through p38 and p44/42 MAP kinase, or nuclear factor kappa- B (NF-κB), and leads to production of reactive oxygen species (ROSs) and pro-inflammatory cytokines production [5, 8]. RAGE is expressed on numerous cell types, including vascular cells, adipocytes, podocytes, immune cells, neurons, cardio-myocytes, and lung epithelial cells [9–12]. RAGE has numerous ligands other than AGEs that can bind to either extracellular V-type immunoglobulin (Ig) domain with numerous special and distinct binding sites or to extracellular C1 and C2-type Ig domains; these further confirm the complexity of RAGE-ligand interactions [13–15].

Soluble receptor for advanced glycation end products (sRAGE) are a particular form of RAGE found in plasma and other fluids of the body, such as synovial fluid and cerebrospinal fluids [16]. There are two major forms of sRAGE; most of the circulating sRAGE results from cell surface-cleavage of the full-length receptor by matrix metalloproteinases (MMPs) [17, 18] and the other less prevalent form of sRAGE, known as endogenous secretory or esRAGE, is a product of a splice variant of AGE receptor (AGER) [19]. Numerous evidence are available about the pathogenic role of AGE and RAGE in inducing inflammation, oxidative stress, adipocyte hypertrophy and expansion, as well as ectopic lipid accumulation in different organs [20–22]. On the other hand, it is suggested that sRAGE, working as a decoy receptor, can bind to RAGE ligands and prevent membrane RAGE activation and associated detrimental health effects [23, 24]. Several studies have investigated the positive effects of sRAGE in prevention of obesogenic effects of AGE and its receptor RAGE. Dozio E et al. [24] reported lower sRAGE concentrations in women with obesity versus women with normal weight (*P* < 0.05). In another study by Zaki M et al. [25], similar finding was reported. Some other studies also reported positive associations between BMI and sRAGE concentrations [26–29]. However, some other studies reported no significant association between BMI and sRAGE [30, 31]. Since the exact association between obesity and sRAGE is not clear, a critical analysis can help to better identify this association. Accordingly, we aimed to quantify and critically review the results of studies reporting the associations between sRAGE with central or general obesity indices in general adult populations.

## Methods and materials

To report the results, we used the Preferred Reporting Items for Systematic Reviews and Meta-Analyses (PRISMA) (Sup. Table 1) [32].

### Eligibility criteria

In the current systematic review and meta-analysis, inclusion criteria were as follows: (1) observational studies with cross-sectional design (2); studies evaluating the relationship between sRAGE and obesity; (3) studies including measurements of general or central obesity including body mass index (BMI), fat mass, waist circumference (WC), or waist to hip ratio (WHR); (4) studies conducted among apparently healthy adults, with obesity or obesity-related disorders including diabetes, cardiovascular diseases, metabolic syndrome, and aged over 18 years; and (5) studies that provided the mean ± standard deviation (SD) of BMI, fat mass, WC, or WHR of those in the lowest versus highest categories of sRAGE. Interventional trials, case-reports and case-series, animal models and in vitro studies, reviews, congress, seminars, letter to editors and short communications were excluded. The PECO (patients, exposure, control/comparator and outcome) model for selecting the studies is presented in Table 1. This model is one of the most widely used models for formulating clinical questions.

**Table 1:** The PECO criteria used for the systematic review

PICO criteria	Description
Participants	Adult population who are apparently healthy, obese, or have obesity-related disorder
Exposure	Highest category of sRAGE
Control/ Comparisons	Lowest category of sRAGE
Outcome	BMI, WC, WHR
Study design	Cross-sectional study

### Information sources, search strategy and selection process

Our systematic search in electronic databases, including Scopus, PubMed and Embase up to 26 October, 2023 yielded a total of 21,612 articles. The search was limited only to English language articles. No missing document was found through hand-searching the reference lists of the papers. The search strategy was created with a combination of the MeSH (Medical Subject Headings) (Sup. Table 2). Two independent reviewers evalutaed the articles for meeting the inclusion criteria. Any problem was resolved by a third reviewer in case of inconsistencies.

### Data collection and extraction process

Four independent researchers performed data extraction, of the following information, including the name of first author, journal, country, publication year, demographic information of participants (e.g., age, gender distribution, and percent of male participants), baseline BMI and WC, study design, sample size, adjusted covariates, study setting, sRAGE measurement tools, and main findings.

### Risk of bias assessment

The methodological quality of included studies for risk of bias assessment were assessed using the Agency for Healthcare Research and Quality (AHRQ) checklist [33, 34]. 

### Synthesis methods

Data analysis was performed by STATA version 16 (STATA Corp, College Station, TX, USA). *P*-values less than 0.05 were considered as statistically significant. The studies that reported the comparison of BMI or WC [mean (SD)] in those with highest versus lowest sRAGE categories were evaluated. Therefore, the mean and SD of variable was used to calculate weighted mean difference (WMD) with 95% confidence interval (CI). When the median and range were reported and mean and SD were not available, the median values were considered as the best estimate of mean and the SD was calculated as the Hozo et al method as below: ${SD}^{2}\approx \left(\frac{1}{12}\right(\frac{{\left(a-2m+b\right)}^{2}}{4}+{\left(b-a\right)}^{2}$) where “SD” is standard deviation, “a” and “b” are upper and lower limits of range, and “m” is the median value [35, 36]. For missing SDs, the method of Walter and Yao was used [37]. Cochran’s Q and I^2^ tests were used for heterogeneity measurements; for statistically significant heterogeneities (e.g., either *P* value for Q statistics of less than 0.1 or I_2_ greater than 50%), random effects model we used [38–40]. To identify the source of heterogeneity, subgrouping and meta-regression approaches were performed. Begg’s Funnel plots, Begg’s correlation coefficient and Egger’s asymmetry tests were used for assessment of publication bias.

## Results

### Study selection

**Fig. 1:**
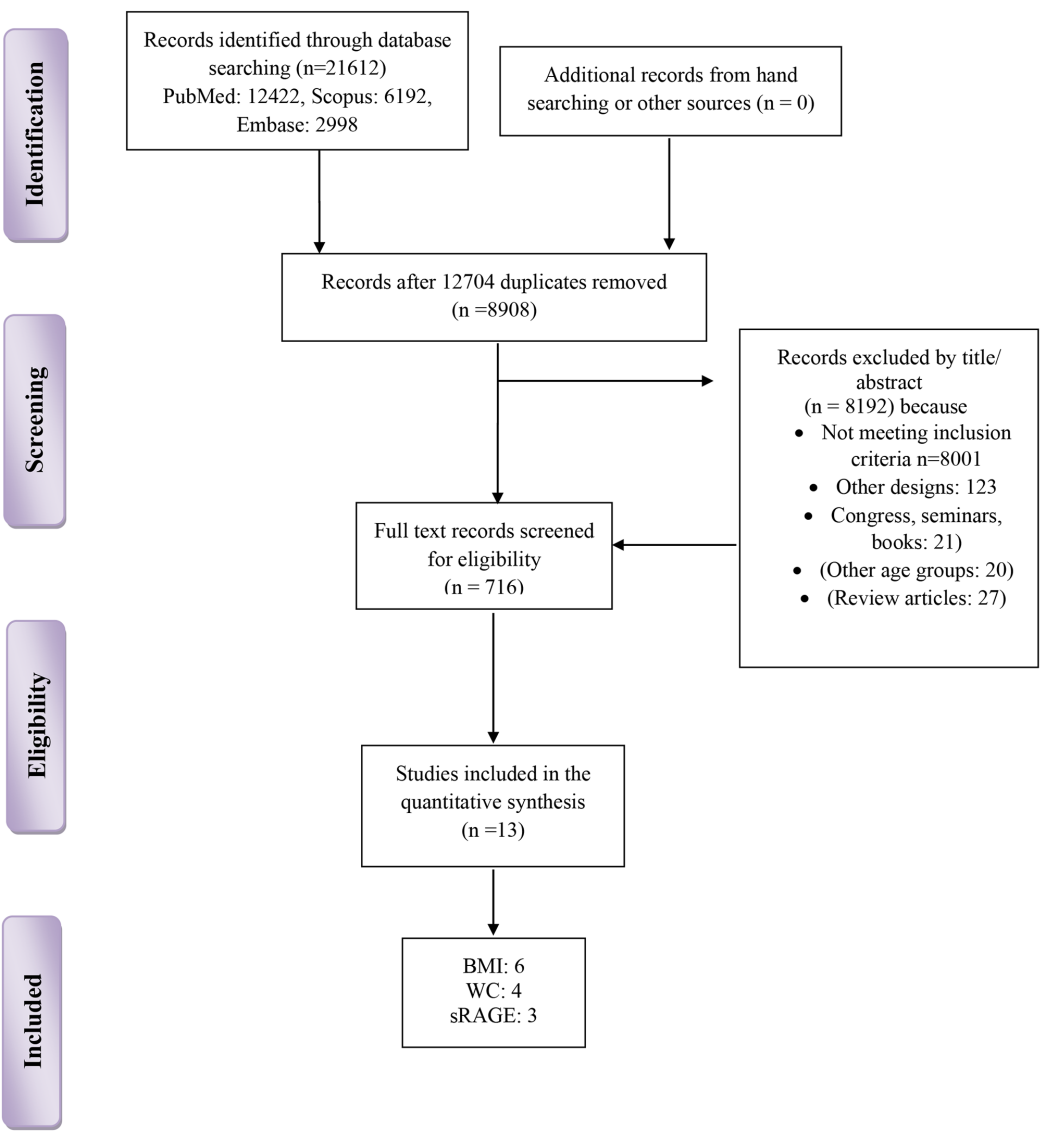
Study flowchart

Our search results yielded a total of 21,612 articles that were imported into the EndNote software. In the first phase, 12,704 articles were removed due to duplication. Next, 8,192 articles were removed after screening the title and abstract due to not meeting inclusion criteria, other designs and age groups, and being seminars, congresses, and review articles. Finally, a total of 716 articles remained for full-text evaluation by two independent reserachers. Consequently, 13 manuscripts were included in the final meta-synthesis. The study’s selection flowchart is presented in Fig. 1.

### Study characteristics

In the meta-analysis of the comparison of BMI between highest versus lowest sRAGE categories, six individual studies with a total number of 1,865 participants were included. General characteristics of these studies are shown in Table 2. Two out of eight studies were performed among obese, diabetic patients [31, 41] and the six other studies [25–28, 30, 42] were performed among apparently healthy population. The age range of study participants was 18–83 years old and BMI was 18–42 kg/m^2^. In the study by Momma H (2014) [26], BMI was significantly lower in the highest versus the lowest category of sRAGE among general Japanese adult population (22.7 versus 24.5 kg/m^2^; *P* < 0.001). Similarly, in another study by the same author [27], BMI was significantly lower in the highest versus the lowest category of esRAGE (21.9 versus 23.3 kg/m^2^; *P* < 0.001). Other studies by Moriya S et al. in general healthy population of Japan [28], by Moy KA et al. in Finland [30], and by Zaki M et al. in Egypt [25] found similar results. Similarly, in the meta-analysis of the comparison of WC between highest versus lowest sRAGE categories, four individual studies with 1,876 participants from the USA [42], Japan [26, 27], and Egypt [25], WC was significantly lower in the highest versus the lowest category of sRAGE in all the studies. The mean sRAGE concentration in serum was compared between individuals with obesity versus individuals without obesity in three individual studies with 165 participants [31, 41]; the individuals with obesity had lower mean sRAGE compared to individuals without obesity.

**Table 2:** Characteristics of the studies included in the systematic review

*Studies that reported the comparison of BMI between the highest versus the lowest sRAGE categories*									
First Author/ year	Country	Journal	Study Population	Gender/ male (%)	Age	BMI (kg/m^2^)	WC (cm)	Num.	Main finding
Momma H/ 2014 [26]	Japan	Diabetol Metab Syndr	Healthy	Both / 80	30–83	22–25	82–86	712	BMI was significantly lower in the highest versus the lowest category of sRAGE (22.7 versus 24.5 kg/m^2^; *P* < 0.001)
Momma H/ 2014 [27]	Japan	J Clin Endocrinol Metab	Healthy	Men/ 100	30–83	21–24	81–84	426	BMI was significantly lower in the highest versus the lowest category of esRAGE (21.9 versus 23.3 kg/m^2^; *P* < 0.001)
Moriya S/ 2014 [28]	Japan	J Stroke Cerebrovasc Dis	Healthy	Both /58	40–60	22–24	-	142	BMI in the highest category of sRAGE was significantly lower than the lowest (22.3 vs. 23.6 kg/m2; *P* < 0.001) Also, BMI in the highest category of esRAGE was significantly lower than the lowest (22.1 vs. 23.7 kg/m^2^; *P* < 0.001)
Moy KA/ 2013 [30]	Finland	Hepatology	Healthy	Men/ 100	50–69	25–30	-	485	BMI in the highest category of sRAGE was non-significantly lower than the lowest (26.4 vs. 27.1 kg/m^2^; *P* = 0.12)
Zaki M/ 2017 [25]	Egypt	Excli J	Healthy	Women/ 0	18–35	25–35	75–100	100	BMI in the highest category of sRAGE was significantly lower than the lowest (24.8 vs. 35.9 kg/m^2^; *P* < 0.05)
***Studies that reported the comparison of WC between highest versus lowest sRAGE categories***									
Chen L/ 2016 [42]	USA	Cancer Epidemiolo	Healthy	Women/ 0	50–79	18–40	75–90	638	WC was significantly lower in the highest versus the lowest category of sRAGE (80.4 versus 86 cm; *P* < 0.001)
Momma H/ 2014 [26]	Japan	Diabetol Metab Syndr	Healthy	Both / 80	30–83	22–25	82–86	712	WC was significantly lower in the highest versus the lowest category of sRAGE (82 versus 86 cm; *P* < 0.001)
Momma H/ 2014 [27]	Japan	J Clin Endocrinol Metab	Healthy	Men/ 100	30–83	21–24	81–84	426	WC was significantly lower in the highest versus the lowest category of sRAGE (81 versus 83 cm; *P* < 0.001)
Zaki M/ 2017 [25]	Egypt	Excli J	Healthy	Women/ 0	18–35	25–35	75–100	100	WC was significantly lower in the highest versus the lowest category of sRAGE (80.7 versus 96.2 cm; *P* < 0.001)
***Studies that reported the comparison of sRAGE between individuals with and without obesity.***									
Amin MN/ 2011 [41]	Egypt	Int J Biomed Sci	Obese apparently healthy	Both /14	46–48	19–50	-	30	Mean sRAGE in individuals with obesity was significantly lower compared with individuals without obesity (660.60 versus 504.42 pg/ml; *P* < 0.05)
Amin MN/ 2011 [41]	Egypt	Int J Biomed Sci	Obese diabetic	Both /14	46–48	19–50	-	58	Mean sRAGE in patients with diabetes and obesity was significantly lower compared with patients with diabetes but without obesity (294.68 versus 333.89 pg/ml; *P* < 0.05)
Davis KE/ 2014 [31]	USA	Nutr Res	Obese apparently Healthy	Both /32	18–45	19–25	-	77	Mean sRAGE in individuals with obesity was non significantly lower compared with individuals without obesity (404 versus 643 pg/ml; *P* = 0.07)

sRAGE, soluble receptor for advanced glycation end products; esRAGE, endogenous secretory receptor for advanced glycation end products; BMI, body mass index; WC, waist circumference; in all of the studies, circulating sRAGE concentrations were measured with enzyme-linked immunosorbent assay (ELISA).

### Risk of bias in the included studies

The results of risk of bias assessment is provided in Table 3 by AHRQ checklist [33]. As shown in this Table, four out of eight studies had moderate quality and the remaining four studies had high quality. The lowest quality score was 5 and the highest quality score was 9.

**Table 3:** Agency for Healthcare Research and Quality (AHRQ) checklist to assess quality of the cross-sectional studies

ARHQ Methodology Checklist items for Cross-Sectional study	Momma H [26]	Momma H [27]	Moriya S [28]	Moy KA [30]	Zaki M [25]	Amin MN [41]	Davis KE [31]	Chen L [42]
1) Define the source of information (survey, record review)	⊕	⊕	⊕	⊕	⊕	⊕	⊕	⊕
2) List inclusion and exclusion criteria for exposed and unexposed subjects (cases and controls) or refer to previous publications	⊕	⊕	⊕	⊕	⊕	⊕	⊕	⊕
3) Indicate time period used for identifying patients	⊕	⊕	⊕	⊕	⊕	⊕	⊕	⊕
4) Indicate whether or not subjects were consecutive if not population-based	⊕	U	⊕	⊕	⊕	U	⊕	⊕
5) Indicate if evaluators of subjective components of study were masked to other aspects of the status of the participants	U	U	U	U	U	U	U	U
6) Describe any assessments undertaken for quality assurance purposes (e.g., test/retest of primary outcome measurements)	**U**	**U**	U	**U**	U	U	U	⊕
7) Explain any patient exclusions from analysis	⊕	⊕	⊕	⊕	⊕	⊕	⊕	⊕
8) Describe how confounding was assessed and/or controlled.	⊕	⊕	U	⊕	U	U	U	⊕
9) If applicable, explain how missing data were handled in the analysis	⊕	⊕	U	⊕	U	U	U	⊕
10) Summarize patient response rates and completeness of data collection	⊕	⊕	⊕	⊕	⊕	⊕	⊕	⊕
11) Clarify what follow-up, if any, was expected and the percentage of patients for which incomplete data or follow-up was obtained	-	-	-	-	-	-	-	-
Total score	8	8	6	8	6	5	6	9

### Results of synthesis

**Fig. 2:**
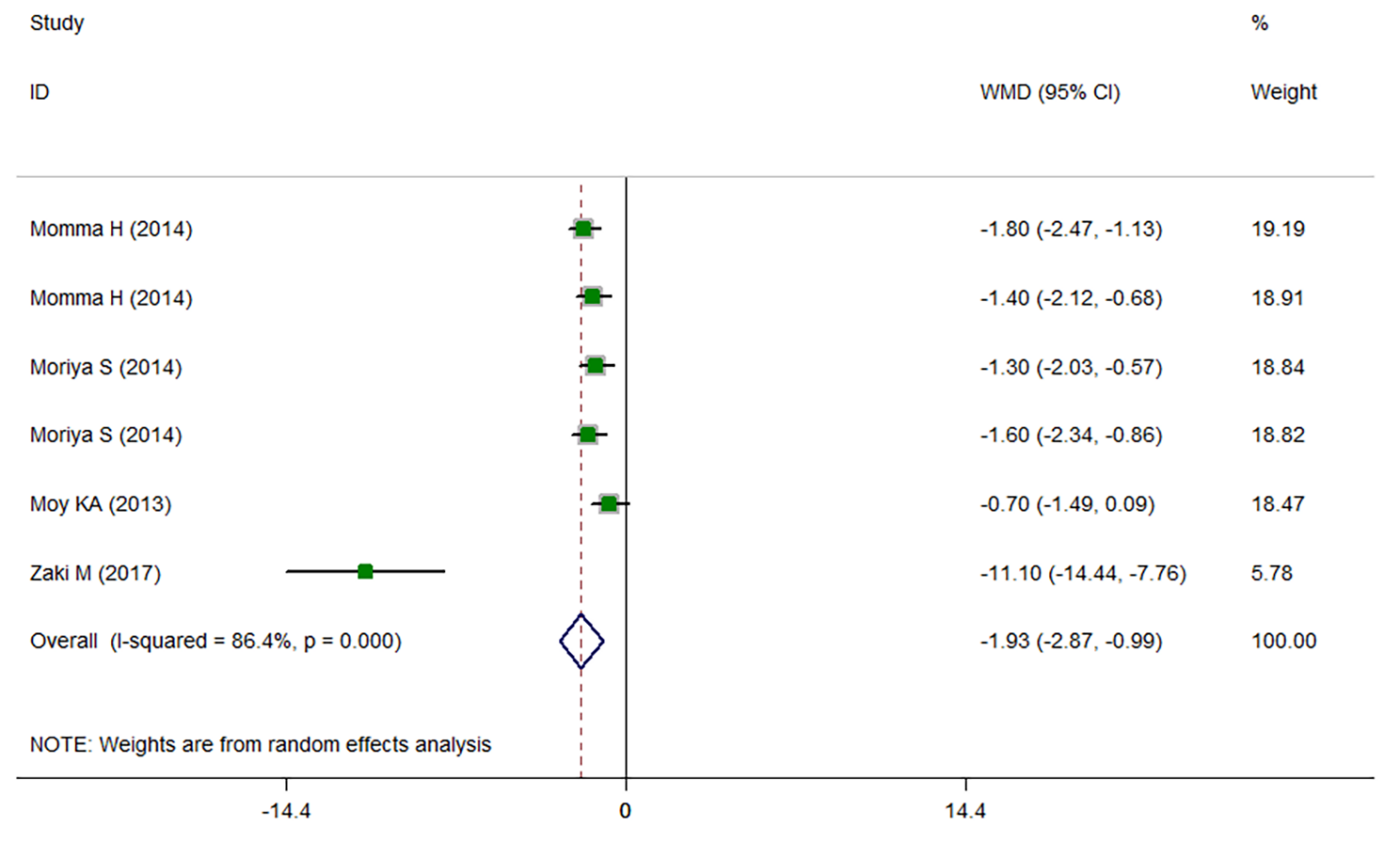
Weighted mean difference (WMD) with 95% confidence interval (CI) of body mass index (BMI) in the highest versus the lowest soluble receptor for advanced glycation end products (sRAGE) categories. I^2^ represents the degree of heterogeneity

**Fig. 3:**
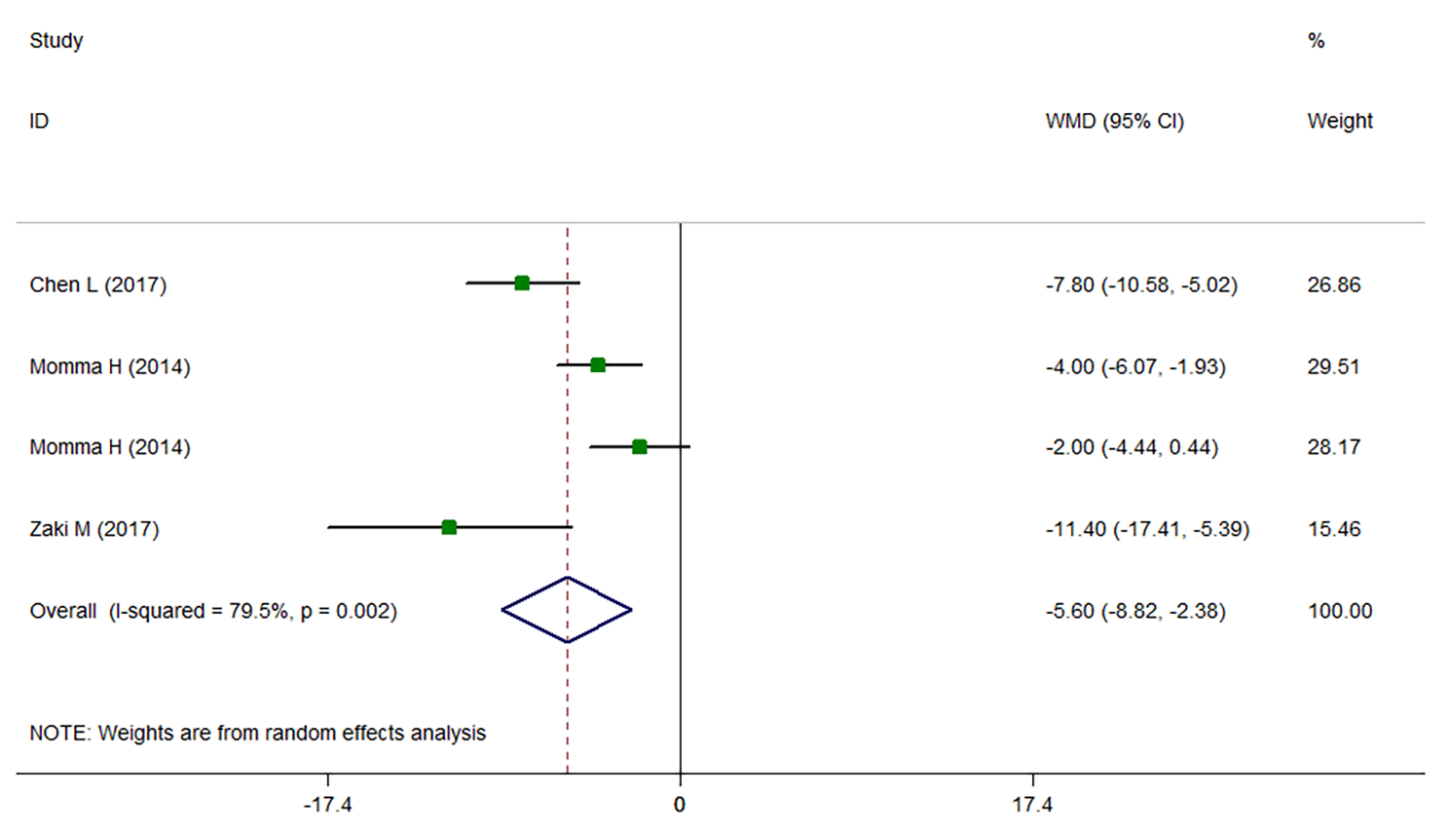
Weighted mean difference (WMD) with 95% confidence interval (CI) of waist circumference (WC) in the highest versus the lowest soluble receptor for advanced glycation end products (sRAGE) categories. I^2^ represents the degree of heterogeneity

**Fig. 4:**
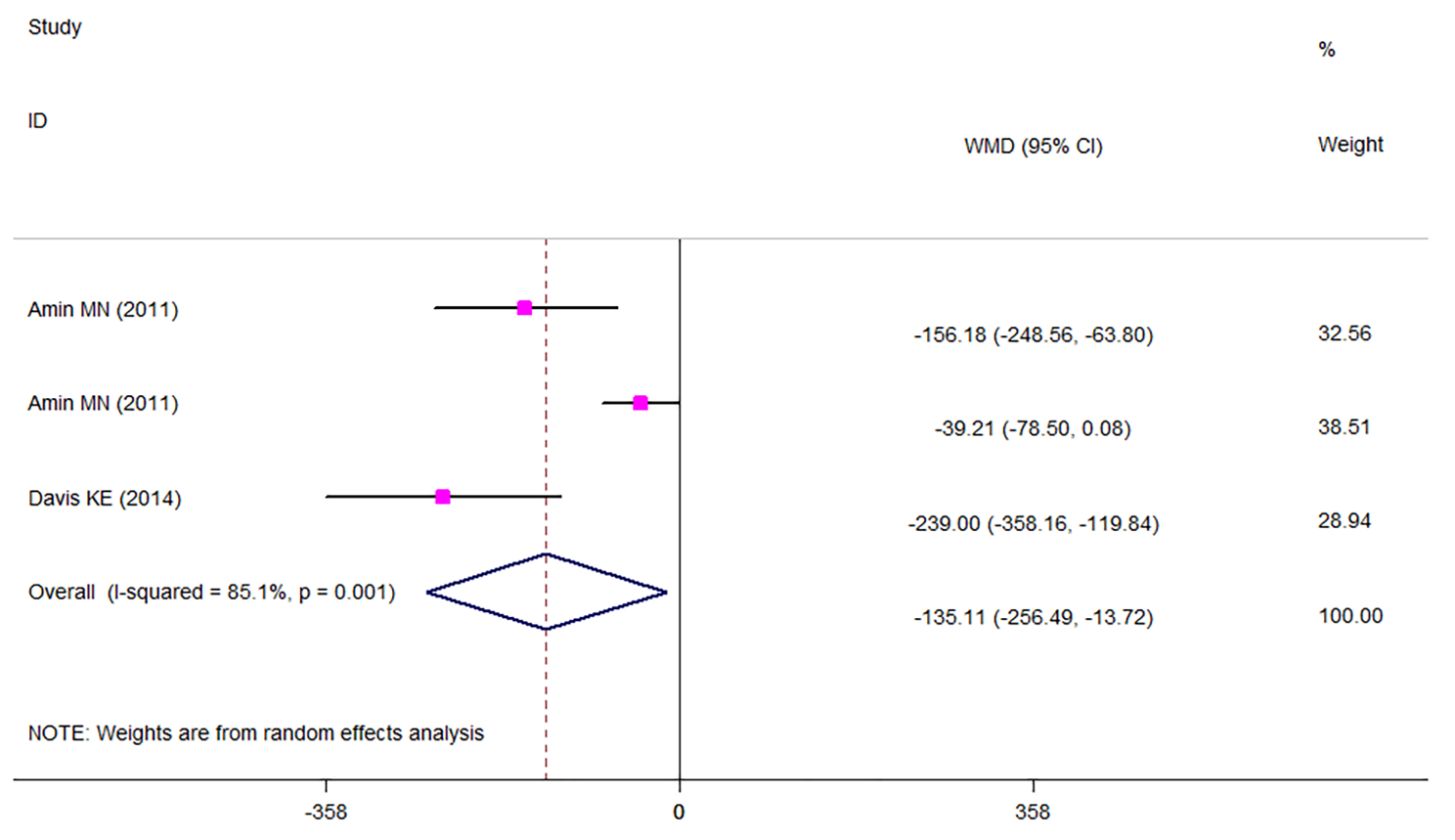
Weighted mean difference (WMD) with 95% confidence interval (CI) of circulating soluble receptor for advanced glycation end products (sRAGE) concentrations in individuals with obesity versus individuals without obesity. I^2^ represents the degree of heterogeneity

The results of meta-analysis (Fig. 2) showed that being at the highest category of sRAGE with median values of 934.92 pg/ml of sRAGE, was associated with lower BMI among apparently healthy adults (WMD: -1.927; CI: -2.868, -0.986; *P* < 0.001) compared with those at the lowest category of sRAGE concentration with median values of 481.88 pg/ml. Similarly, those at the highest sRAGE category with the median values of 1302.3 pg/ml sRAGE had about 6 cm lower WC compared with those at the lowest category of sRAGE concentration with median values of 500.525 pg/ml (WMD: -5.602; CI: -8.820, -2.383; *P* < 0.001; Fig. 3). Comparing the sRAGE concentrations in individuals with obesity versus individuals without obesity, individuals with obesity had significantly lower circulating sRAGE concentrations (WMD: -135.105; CI: -256.491, -13.72; *P* = 0.029; Fig. 4). Subgrouping results of the comparison of BMI between the highest versus the lowest category of sRAGE are presented in Table 4. In studies performed in Japan that had baseline BMI of lower than 23 kg/m^2^, initial heterogeneity reduced from 86.4 to 0. Therefore, country and baseline BMI might be the sources of heterogeneity. The results of subgrouping for the comparison of WC between sRAGE categories are shown in Table 5. Accordingly, country might be a heterogeneity source. However, interpretation of results is challenging due to the low number of studies in each subgroup. The results of meta-regression (Tables 6 and 7) also confirmed these findings. The results of funnel plots (Sup. Figures 1, 2) and Begg’s adjusted rank correlation and Egger’s regression asymmetry tests showed no publication bias (for BMI; Egger *P*-value: 0.224; Begg’s *P*-value: 0.851 and for WC: Egger *P*-value: 0.497; Begg’s *P*-value: 0.297).

**Table 4:** Subgroup analysis for the comparison of BMI between the highest versus the lowest category of sRAGE

Group	No. of studies^*^	WMD (95% CI)	P _within group_	P _between group *_	P _heterogeneity_	I^2^, %
**Total**	6	-1.927 -2.868 -0.986	< 0.001	< 0.001	< 0.001	86.4
**Country**						
*Japan*	4	-1.536 -1.893 -1.180	< 0.001		0.763	0
*Others*	2	-5.768 -15.957 4.420	0.267		< 0.001	97.2
***Baseline BMI (kg/m***^***2***^)				< 0.001		
*23 >*	4	-1.536 -1.893 -1.180	< 0.001		0.763	0.0
*≥ 23*	2	-5.768 -15.957 4.420	0.267		< 0.001	97.2
**RAGE type**				< 0.001		
*sRAGE*	4	-2.591 -4.225 -0.956	0.002		< 0.001	91.8
*esRAGE*	2	-1.498 -2.013 -0.983	< 0.001		0.703	0.0
**Gender**				< 0.001		
Men	2	-1.614 -2.106 -1.123	< 0.001		0.426	0.0
Women	1	-11.100 -14.441 -7.759	< 0.001		-	-
Both	3	-1.219 -1.726 -0.712	< 0.001		0.257	26.5
**Age group**				< 0.001		
*< 80*	3	-1.329 -1.946 -0.712	< 0.001		0.116	53.7
*< 60*	2	-1.450 -1.968 -0.931	< 0.001		0.571	0.0
*< 40*	1	-11.10 -14.441 -7.759	< 0.001		-	-
***Sample size***				< 0.001		
*300 >*	3	-3.655 -6.095 -1.215	0.003		< 0.001	93.7
*≥ 300*	3	-1.329 -1.946 -0.712	< 0.001		0.116	53.7
***Study quality***				< 0.001		
*Moderate*	3	-3.655 -6.095 -1.215	0.003		< 0.001	93.7
*High*	3	-1.329 -1.946 -0.712	< 0.001		0.116	53.7

All the included studies had a cross-sectional design

**Table 5:** Subgroup analysis for the comparison of WC between the highest versus the lowest category of sRAGE

Group	No. of studies^*^	WMD (95% CI)	P _within group_	P _between group *_	P _heterogeneity_	I^2^, %
**Total**	4	-5.602 -8.820 -2.383	0.001		0.002	79.5
**Country**				< 0.001		
*Japan*	2	-3.109 -5.057 -1.161	0.002		0.221	33.3
*Others*	2	-8.572 -11.468 -5.676	< 0.001		0.287	11.8
**Baseline WC (cm)**				< 0.001		
*82 >*	2	-4.860 -10.543 0.823	0.094		0.002	89.4
*≥ 82*	2	-7.139 -14.307 0.029	0.051		0.023	80.8
**RAGE type**				< 0.001		
*sRAGE*	3	-7.005 -10.774 -3.237	< 0.001		0.016	75.7
*esRAGE*	1	-2.000 -4.441 0.441	0.108		-	-
**Gender**				< 0.001		
Men	2	-3.109 -5.057 -1.161	0.002		0.221	33.3
Women	1	-11.40 -17.415 -5.385	< 0.001		-	-
Both	1	-7.800 -10.583 -5.017	< 0.001		-	-
**Age group**				< 0.001		
*< 80*	3	-4.523 -7.578 -1.469	0.004		0.008	79.1
*< 40*	1	-11.40 -17.415 -5.385	< 0.001		-	-
***Sample size***	2					
*500 >*	2	-6.282 -15.457 2.894	0.180	< 0.001	0.005	87.6
*≥ 500*	2	-5.782 -9.498 -2.065	0.002		0.032	78.3
***Study quality***				< 0.001		
*Moderate*	1	-11.40 -17.415 -5.385	< 0.001		-	-
*High*	3	-4.523 -7.578 -1.469	0.004		0.008	79.1

All the included studies had a cross-sectional design.

**Table 6:** Meta regression analysis for finding the possible sources of heterogeneity for the association between sRAGEs and BMI

sRAGEs			
	Tau^2^	P	95%CI
**Estimate of between-study variance**	1.0839		
By region (Japan versus others)	-3.968534	0.293	-13.08245 5.145382
By sample size (> 300 versus others)	1.449087	0.634	-6.359169 9.257342
By study quality (high versus others)	-2.712364	0.133	-6.603796 1.179068
By gender (both versus others)	1.075548	0.733	-7.074379 9.225475
By age (< 80 versus others)	1.449	0.634	-6.359169 9.257342
By baseline BMI (< 23 versus others)	-3.968534	0.293	-13.08245 5.145382
By sRAGEs type (sRAGE versus esRAGE)	2.030782	0.513	-5.830596 9.892159

sRAGEs, Soluble receptor for advanced glycation end products; BMI, body mass index; CI, confidence interval

**Table 7:** Meta regression analysis for finding the possible sources of heterogeneity for the association between sRAGEs and WC

sRAGEs			
	Tau^2^	P	95%CI
**Estimate of between-study variance**	8.0213		
By country (Japan versus others)	-7.489708	0.940	-387.5201 372.5407
By sample size (> 500 versus others)	-1.632441	0.987	-380.3538 377.089
By study quality (high versus others)	-5.712109	0.060	-11.88682 0.4626038
By gender (Male versus others)	-7.489708	0.940	-387.5201 372.5407
By age (< 80 versus others)	-10.77336	0.930	-476.9407 455.394
By baseline WC (> 82 versus others)	5.166737	0.959	-374.1479 384.4813
By sRAGEs type (sRAGE versus esRAGE)	6.775087	0.951	-414.3968 427.9469

sRAGEs, soluble receptor for advanced glycation end products; esRAGE, endogenous secretary receptor for advanced glycation end products; WC, waist circumference; CI, confidence interval

## Discussion

**Fig. 5:**
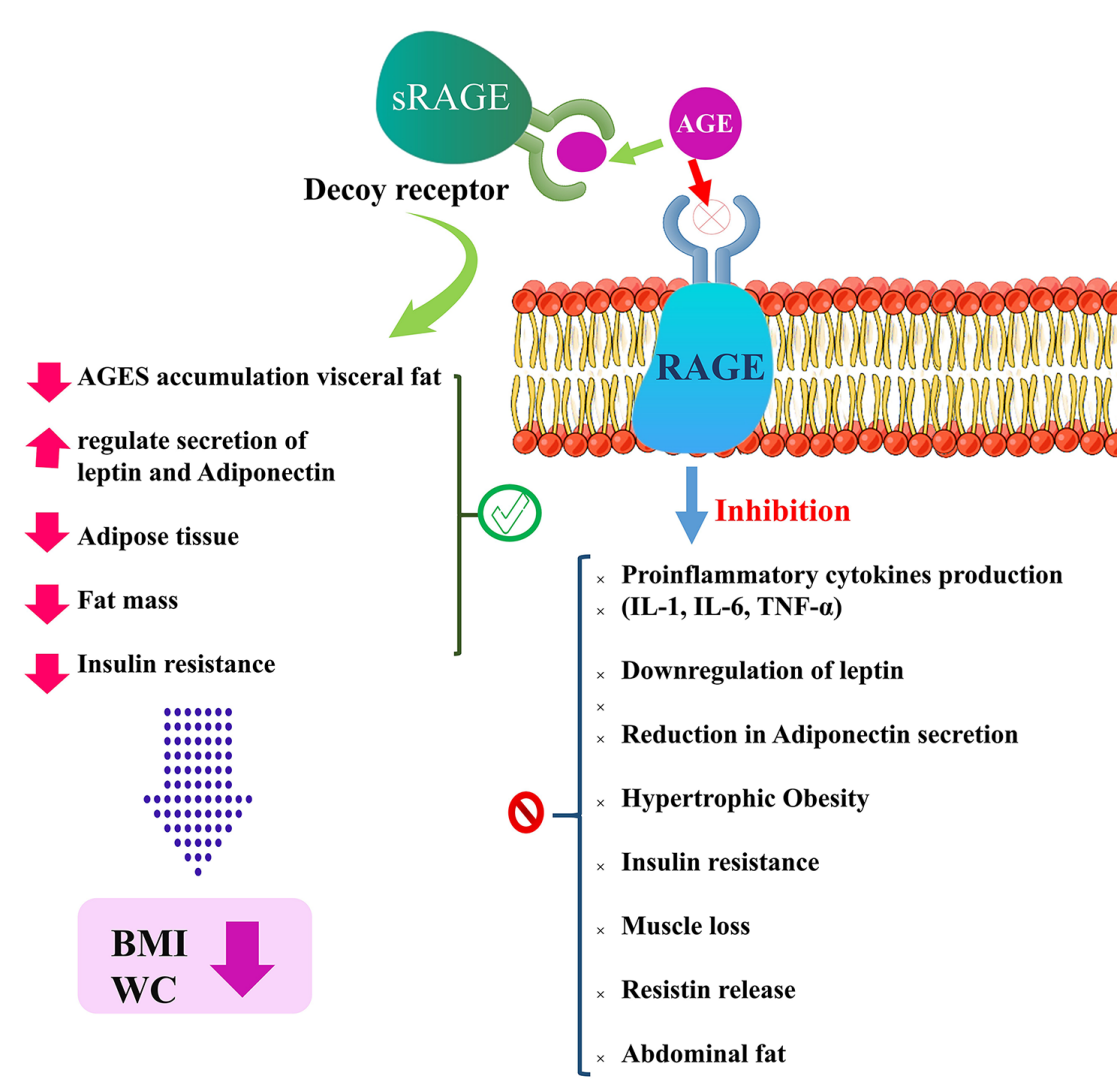
The accumulation of AGEs in adipocytes may explain the inverse association between AGE levels and BMI; high-AGE diets are associated with greater fat consumption and an increased risk of abdominal obesity [55]. RAGE activation also raises circulating inflammatory cytokines while decreasing anti-inflammatory adipokines like adiponectin. IL-6 and leptin are recognized as inflammatory mediators in the presence of obesity [56]. Moreover, adipocyte hypertrophy is associated with enhanced RAGE expression [57]. However, the involvement of sRAGE in the prevention of age-related muscle mass loss must be addressed [58]. As a decoy receptor for AGEs and other inflammatory ligands, sRAGE attributed to the reduced ligand-mediated disruption, preventing AGEs from binding to the cell-bound full-length receptor RAGE [58] and inhibiting the obesogenic effects of AGE-RAGE interaction by diminishing AGES accumulation visceral fat, regulating leptin and Adiponectin release [56], reducing Insulin resistance [58]. AGEs = advanced glycation end products; RAGE = receptor for advanced glycation end products; sRAGE = soluble receptor for advanced glycation end products; IL-1 = interleukin-1; IL-6 = interleukin-6; TNF-α = Tumor Necrosis Factor alpha; BMI = Body mass index; WC = Waist Circumference

In the current meta-analysis, higher sRAGE concentrations was associated with lower BMI among 1,865 apparently healthy individuals. Also, lower WC was accompanied with higher sRAGE concentrations among 1,876 adults. This is the first meta-analysis evaluating the association between sRAGE and obesity indices, and reporting obesity-reducing effects of sRAGE among adults. There are several underlying mechanisms suggested for obesity prevention by sRAGE, such as protection against obesity-induced lipid accumulation by preventing RAGE hyper-expression [43]. Also, it is suggested that higher AGE flux in individuals with obesity reduces sRAGE levels; it is well-known that sRAGE has other ligands besides AGEs that could decrease it. So, a lower sRAGE among individuals with obesity is a reflection of greater binding of AGEs to its ligands [44, 45]. Since numerous studies revealed lower circulating AGEs concentrations in individuals with obesity, one interesting finding is that, possibly, CML concentrations are not a good biomarker of its status in individuals with obesity, and further studies are needed to confirm whether it is the case for different AGE compounds [46–49]. This might be attributed to the fact that the most preferable AGEs measured in the studies is CML, and it is suggested that circulating CML concentrations is in inverse association with body fat storage, because it deposits in fat tissue and by fat mass expansion (like in individuals with obesity) its deposition in fat mass increases and lowers its circulating amount [48, 50, 51]. As a result, circulating sRAGE values would be a better marker of the AGE-RAGE interaction in the body and its lower concentrations is a reflection of detrimental effects of this interaction. Impaired adipocyte function is also suggested as possible mechanism of increased central obesity in lower sRAGE concentrations [26, 52]. The possible underlying mechanisms of the effects of AGE-RAGE interaction in developing obesity and the protective role of sRAGE is presented in Fig. 5.

In our study, all the included studies had a moderate or high quality, and no study had poor quality. In the subgrouping, geographical area and baseline BMI were the possible sources of heterogeneity. However, the current meta-analysis has some limitations. First, causal inference was not possible due to cross-sectional design of the included studies. Second, due to the low number of studies in each subgroup, making a reliable conclusion is not possible. Third, as the adipose tissue differs between men and women [53, 54], we needed separate data in this regard; however, there was no separate data to evaluate the association of sRAGE levels with BMI or WC by gender.

As a conclusion, in the current systematic review and meta-analysis, for the first time, we quantified and critically reviewed the studies that evaluated the association between general or central obesity indices with circulating sRAGE levels. There was a negative association between BMI, WC, and circulating sRAGE concentrations among adults. Further studies are warranted to confirm our results.

### Electronic supplementary material

Below is the link to the electronic supplementary material.

**Supplementary Material 1: Sup. Table 1**. PRISMA Checklist. **Sup. Table 2**. Search strategies and the number of records according to different electronic database. **Sup. Figure 1**. Begg’s funnel plot (with pseudo 95% CIs) of the weighted mean difference (WMD) versus the standard error (se) of (WMD) for the comparison of (A) body mass index (BMI), (B) waist circumference (WC) in those of the highest versus lowest soluble receptor for advanced glycation end products (sRAGE) categories [BMI: P egger= 0.224; P begg =0.851; WC, P egger= 0.297; P begg =0.497]

## Data Availability

The datasets generated and/or analyzed in the current study are not publicly available due to some restrictions applied by the ethics committee. However, they are available from the corresponding author on reasonable request.
